# Poorly differentiated mucinous carcinoma with signet ring cells in an ovarian endometriotic cyst: a case report

**DOI:** 10.1186/s13000-019-0850-0

**Published:** 2019-07-06

**Authors:** Yurong Jiao, Bingjian Lu

**Affiliations:** 1 Department of Surgical Pathology, Women’s Hospital, School of Medicine, Zhejiang University, Hangzhou, Zhejiang Province China; 2 Center for Uterine Cancer Diagnosis & Therapy Research of Zhejiang Province, Women’s Hospital, School of Medicine, Zhejiang University, Hangzhou, Zhejiang Province China

**Keywords:** Endometriosis, Ovary, Primary mucinous carcinoma, Signet ring cell

## Abstract

**Background:**

Ovarian signet ring cell carcinomas are predominantly metastatic. Cases coexisting with endometriotic cysts are extremely rare, and are supposed to be primary. However, such cases have not been well-documented to date.

**Case presentation:**

A 46-year-old Chinese woman had an incidental small nodule in the right ovarian endometriotic cyst. She underwent a staging surgery due to an unexpected ovarian carcinoma from her frozen section. Laparotomy exploration, MRI and gastrointestinal endoscopy revealed no other abnormalities in the abdominal organs. She had a pelvic recurrence at 7 months and was alive with disease for 13 months at present. Gross examination showed a small mural nodule (l.0 × 0.5 × 0.2 cm) in the wall of the right ovarian cyst (18x15x14 cm). Microscopically, the neoplastic cells arranged in solid nests, crowded small irregular glands and scattered single cells. They had abundant cytoplasmic mucin and contained a significant component of signet ring cells. The stroma was desmoplastic and occasionally contained extracellular mucin deposits. The surrounding endometriotic cyst had several foci of atypical surface epithelium (atypical endometriotic cyst) that was continuous with the mucinous carcinoma. Immunohistochemistry demonstrated that the tumor cells were diffusely positive for CK7 and negative for CK20 and CDX2.

**Conclusions:**

Primary ovarian poorly differentiated mucinous carcinoma with signet ring cells can occur in an atypical endometriotic cyst. This rare case addresses the necessity of careful and extensive pathological examination on large ovarian endometriotic cysts.

## Background

Endometriosis is a common benign disease in women. It occurs in 3–10% and 2–5% of the reproductive and postmenopausal women, respectively. The ovary is the most common site of endometriosis. Endometriosis-associated ovarian cancer (EAOC) accounts for 0.3–1.0% of endometriosis [1]. The histologic types of EAOCs are predominantly endometrioid and clear cell carcinoma, and rarely serous and mucinous carcinoma [2]. Primary ovarian signet ring cell carcinoma is very unusual, and its relationship with endometriosis has not been well documented yet. In this study, we describe an unusual case of a patient with ovarian mucinous carcinoma containing a significant portion of signet ring cells in an atypical endometriotic cyst.

## Case presentation

### Clinical summary

A 46-year-old Chinese woman, gravida 1 para 1, presented with lower abdominal pain for 5 days and dysmenorrhea for 2 years. Trans-vaginal ultrasound, magnetic resonance image (MRI) and computerized tomography (CT) indicated the presence of uterine adenomyosis and bilateral ovarian endometriotic cysts. There were no abnormalities were found in her bilateral lungs and other abdominal/pelvic organs. The preoperative serum CA-125, carcinomatous embryonic antigen (CEA), and CA-153 were 263.1 U/mL (normal < 35 U/mL), 13.9 U/mL (normal< 5 U/mL) and 33.1 U/mL (normal < 25 U/mL), respectively. She denied her personal and familial history of any cancers and related diseases.

After admission, an abdominal cystectomy of the right ovary was initially performed. The intraoperative frozen section was carried out. Unexpectedly, the frozen section showed an ovarian adenocarcinoma in the wall of the endometriotic cyst. The laparotomy exploration found no abnormalities in other abdominal/pelvic organs including stomach, colon, rectum, appendix, pancreas, and liver. There were no any visible tumorlets in the abdominal/pelvic cavity. The patient eventually underwent a total abdominal hysterectomy with bilateral salpingo-oophorectomy, pelvic and para-aortic lymphadenectomy and omentectomy. The patient was eventually assessed as a stage IC ovarian carcinoma because the tumor partly adhered to the surrounding organs. She recovered smoothly from her surgery. She received 5 courses of TP (paclitaxel + cisplatin) chemotherapy, but she had to abandon her last chemotherapy because of the severe myelo-suppression.

The patient was followed up regularly in our outpatient clinics. The serum CA-125, CEA and CA-153 gradually returned to normal levels within 22 days after her surgery. However, her serum CEA began to elevate (6.3 U/mL) at 6 months after her surgery and rose up to 62.1 U/mL at 7 months. MRI showed a right pelvic mass measuring 9.5*5.7 cm and no abnormalities in the abdominal organs. The patient was transferred to a tertiary hospital for further treatment. The gastrointestinal endoscopic examination with multiple biopsies showed no abnormalities in the stomach, terminal ileum, colon and rectum. A relaparotomy was carried out. The intraoperative findings included a large tumor adhering to the right pelvic wall and the ileum wall, and multiple small grey nodules along the small intestinal mesentery. Other abdominal organs, such as stomach, colon, appendix, pancreas and liver, looked unremarkable. The pelvic tumor and partial ileum were removed. The patient refused to adjuvant therapies including chemotherapy and radiotherapy. She was alive with disease for13 months after her first surgery at present.

### Pathological examination

Grossly, the right ovarian cyst for frozen section measured 18 × 15 × 14 cm. It had a smooth outer surface with occasional rough areas consistent with the adhesions to the surrounding organs. The unilocular cyst was filled with thick, chocolate-like fluids. The inner cystic wall was uneventful except the attachment of some blood clots. There was a small mural nodule with a size of l.0 × 0.5 × 0.2 cm protruding into the lumen. The nodule had a white and solid cut surface. The texture was soft. The right ovary was partly hemorrhagic due to the cystectomy. The left ovary measured 5.0 × 5.0 × 4.5 cm. It had some cystic areas containing dark brown hemorrhagic materials. The uterus measured 10*9*5 cm. The myometrium of the posterior wall was thickened. The cut surface was trabeculated and contained hemorrhagic foci. The endometrium measured 0.2–0.3 cm and looked unremarkable. The uterine cervix and bilateral fallopian tubes were grossly normal.

**Fig. 1:**
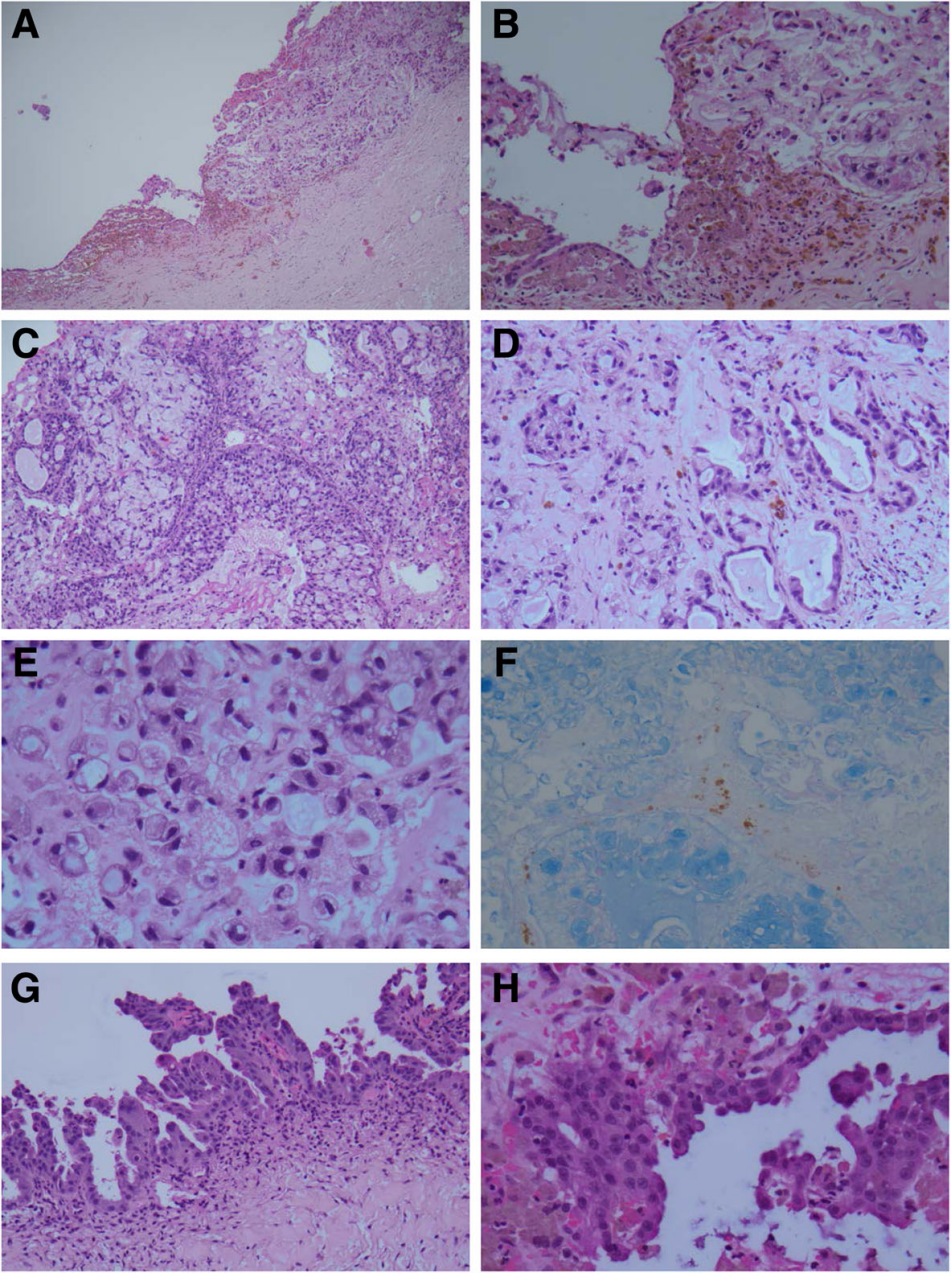
Histopathology of the poorly differentiated mucinous carcinoma with signet ring cells of the ovary. The small carcinomatous nodule is continuous with the epithelium of the endometriotic cyst [**a**, **b**]. The tumor cells arranged in solid nests with occasional cribriform structure and crowded small neoplastic glands with marked nuclear atypia [**c**, **d**]. Signet ring cell carcinoma can be frequently seen [**c**, **e**]. The cytoplasmic mucin of the tumor cells is Alcian Blue positive [**f**]. Atypical surface epithelium in the endometriotic cyst exhibits cellular stratification, small papillation and variable cytological atypia [**g**, **h**]. Original magnifications: **a** × 50; **b**, **d**, **f**, **g** × 200; **c** × 100; **e**, **h** × 400

The mural nodule was histologically continuous with the endometriotic epithelium [Fig. 1a, b]. It showed an invasive growth pattern that were characterized by solid nests with occasional cribriform structures, crowded small irregular glands in sheets and scattered single cells [Fig. 1c, d]. The neoplastic cells in solid nests or in single harbored a typical signet-ring appearance as large mucinous vacuoles and atypical crescentic nuclei [Fig. 1c, e]. The glands were lined by columnar cells with abundant cytoplasmic mucin, significant nuclear atypia, and occasional mitotic figures. The intracellular mucin was Alcian Blue (pH 2.5) positive [Fig. 1f]. The stroma was desmoplastic and had occasional extracellular mucin deposits. There was no lymphovascular invasion or surface involvement of the ovary. The remaining cystic wall showed a typical morphology of an endometriosis that was composed of endometrial epithelium and stroma with hemosiderin-laid macrophages [Fig. 1a, b]. The epithelium adjacent to the tumor showed atypical features (“atypical endometriotic cyst”) that were characterized by cellular crowding, stratification, small papillary formation with eosinophilic cytoplasmic changes, and variable cytological atypia with increased nuclear to cytoplasmic ratio, enlarged hyperchromatic nuclei, and conspicuous nucleoli [Fig. 1g, h]. The surface epithelium was cytologically bland in most areas.

**Fig. 2:**
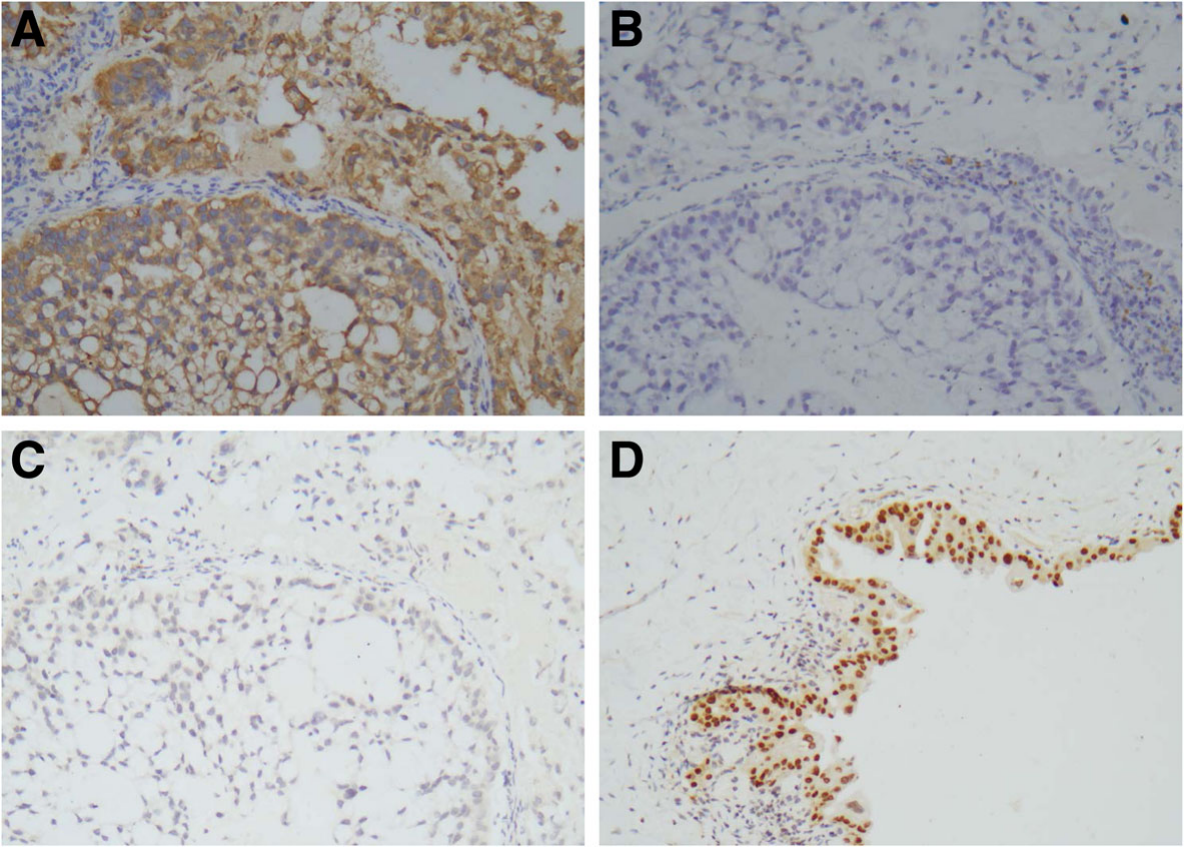
Immunostaining of the poorly differentiated mucinous carcinoma with partial signet ring cell carcinoma of the ovary. The tumor cells are diffusely positive for CK7 [**a**], and negative for CK20 [**b**], PAX-8 [**c**]. The atypical endometriotic epithelium was positive for PAX-8 [**d**]. Original magnifications × 200

Immunohistochemistry demonstrated that the tumor cells were strongly and diffusely positive for CK 7 [Fig. 2a], CEA and p16, and focally positive for CA125, MUC-6 and p53 (DO-7). They were negative for phosphate and tension homology deleted on chromosome ten (PTEN), estrogen receptor (ER), progesterone receptor (PR), CK19, CK20 [Fig. 2b], PAX-8 [Fig. 2c], and CDX2. They did not show loss of hMLH1 and hMSH2 expression. The atypical endometriotic epithelium was positive for PAX-8 [Fig. 2d]. The final pathological diagnosis of the right ovary was poorly differentiated mucinous carcinoma with signet ring cells and concurrent endometriotic cyst with atypical features.

Other pathological findings included endometriotic cysts of the left ovary and uterine adenomyosis. The endometrium showed proliferating-phase alterations. The remaining right ovary, bilateral fallopian tubes, and uterine cervix were pathologically unremarkable. The omentum and pelvic lymph nodes were free of tumor.

The gross feature of the recurrent tumor from her second operation was unavailable in detail. Pathological review of the slides indicated that the recurrent tumor was consisted of poorly-differentiated adenocarcinoma in the fibrous stroma resembling to the original ovarian carcinoma. The tumor involved the outer intestinal muscular proper, but the intestinal mucosa remained normal after careful and extensive sampling. The excision margins were free of tumor.

## Discussion

Primary ovarian mucinous carcinoma is uncommon. It is rarely associated with endometriosis [2]. Ovarian mucinous carcinomas commonly exhibit an expansile or confluent invasion while the traditional destructive stromal infiltration should raise the concern for a metastatic carcinoma. Primary ovarian signet-ring cell carcinoma can occur in the context of teratomas [3], but it is exceptionally rare. Therefore, signet-ring cell carcinoma is highly suggestive of a metastatic neoplasm (Krukenberg tumor). The most likely primary sites include the upper or lower gastrointestinal tract, appendix, the pancreas or biliary tree, and sometimes breast [4–7]. However, Simons et al. [5] addressed that the careful integration of morphology, immunohistochemistry, and clinical and imaging data was required for the conclusive diagnosis of a primary or metastatic ovarian mucinous carcinoma.

Great concerns for a metastatic tumor should be critically posed for the current case, but our comprehensive clinicopathological analysis indicates that the tumor is an ovarian primary most likely. The patient denied any cancer histories. She had no clinical evidence of other primary cancers by now. The gastrointestinal endoscopic examination and biopsies did not show any evidence of malignancy in the stomach, terminal ileum, and colorectum. The CT and MRI in her first admission did not find any abnormalities in her lungs and abdominal/pelvic organs. Her recent MRI only revealed a recurrent tumor in the right pelvic wall. Laparotomy exploration did not have any significant findings in the stomach, colon, liver and pancreas, etc. Primary adenocarcinoma of small intestine was not considered after careful inspection of the resected intestine exhibiting macroscopically and microscopically normal-looking mucosa and negative excision margin. Intriguingly, her serum CEA level increased significantly before operation, returned to normal level gradually after operation and, and elevated with tumor recurrence. The fluctuation of serum CEA does not support the persistence of primary cancers other than the ovary.

A secondary ovarian mucinous carcinoma harbors some common pathological changes including bilateral tumors, a small size, a multiple nodular appearance, ovarian surface implants, extraovarian spread, and extensive lymphovascular invasion [8]. In our patient, most of these pathological changes except the small tumor size are lacking whereas other pathological features, such as the unilateral location, low tumor stage, and background of endometriosis are in support of a primary tumor as reported previously [9]. Immunohistochemistry can aid in the discrimination between a primary and secondary ovarian mucinous tumor. Primary ovarian mucinous tumors are invariably diffusely positive for CK7, and negative or focal positive for CK20 and CDX2 while metastatic mucinous carcinomas, particularly those from the lower gastrointestinal tract and appendix, demonstrate the opposite immunoprofile [10]. The diffuse CK7 positive and CK20 and CDX2 negative immunostaining pattern also supported a primary ovarian mucinous carcinoma.

As early in 1925, Sampson [11] proposed the stringent diagnostic criteria for an ovarian tumor originating from endometriosis for the first time: “*(1) there must be clear endometriosis in proximity to the tumor; (2) no other primary site for the tumor can be found; (3) the presence of tissue resembling endometrial stroma surrounding epithelial glands*.” Scott [12] subsequently suggested the requirement of histological transition or direct relationship between endometriosis and carcinoma. Accordingly, our patient has morphological evidence to support the endometriotic origin by exhibiting the continuity between mucinous carcinoma and the endometriosis, and the presence of multifocal atypical epithelium in the endometriotic cyst. Atypical endometriosis appears to be a possible early precursor in the transition from benign endometriosis to carcinoma despite that the histopathological criteria have not been well established. The suggestive features in atypical endometriosis frequently included eosinophilic cytoplasm, large hyperchromatic or pale nuclei with moderate to marked pleomorphism, an increased nuclear to cytoplasmic ratio, cellular crowding and stratification or tufting [13, 14].

Only 5 primary signet ring cell carcinomas have been described in the English literatures [4, 9, 15, 16]. Some are supposed to be arisen from ovarian teratomas. To the best of knowledge, ovarian endometriosis-associated poorly differentiated mucinous carcinomas with signet ring cells have not been well documented to date. Large endometriotic cysts exceeding 15 cm in diameter are more likely to contain a concurrent neoplasm; thereof, both clinicians and pathologists should be alert to this possibility and to eliminate the missing risk by extensive sampling and meticulous examination. There is no standard treatment for a primary ovarian signet-ring cell carcinoma owing to its rarity. Chemotherapy has been recommended partly due to the high-grade histology and aggressive clinical behavior of their counterparts in the gastrointestinal tract. At least one patient with primary ovarian signet ring cell carcinoma died 5 months after her staging surgery [9] while 3 patients survived without evidence of disease with an interval of 8 months,1 and 3 years, respectively [4, 15]. The early pelvic relapse in our patients may be associated with the adhesion of the ovarian tumor with the surrounding organs, and more importantly, predicts the aggressive clinical course of the poorly differentiated adenocarcinoma.

## Conclusions

In this study, we reported a rare case of primary ovarian poorly-differentiated mucinous carcinoma with signet ring cells in an atypical endometriotic cyst. Our case adds some evidence to support that atypical endometriosis might be an intermediate state in the transition from benign endometriosis to carcinoma. However, accumulative clinicopathological data and molecular analysis are permanently required to consolidate this hypothesis. Finally, the small malignant mural nodule addresses the necessity of careful and extensive pathological examination on large ovarian endometriotic cysts.

## Data Availability

Not applicable.

## Abbreviations

CEA: Carcinomatous embryonic antigen
EAOC: Endometriosis-associated ovarian cancer
ER: Estrogen receptor
PR: Progesterone receptor
PTEN: Phosphate and tension homology deleted on chromosome ten
TP: Paclitaxel + cisplatin
